# Supplementary material for: Pharmacological Modulation of the Unfolded Protein Response as a Therapeutic Approach in Cutaneous T-Cell Lymphoma
Source: Biomolecules. 2025 Jan 7;15(1):76. doi: 10.3390/biom15010076 (PMC11763779; doi:10.3390/biom15010076)
Supplement: Supplementary file 1 [file biomolecules-15-00076-s001.zip › biomolecules-3342041-supplementary.pdf]

# Supplementary Materials

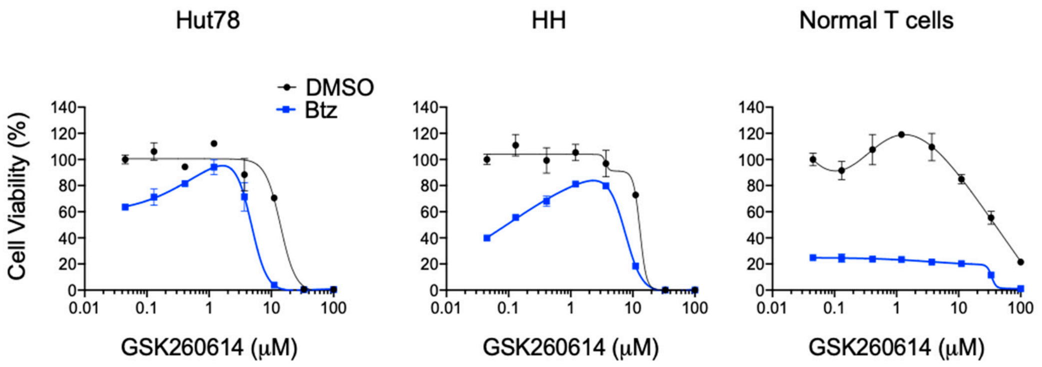


**Figure S1. Btz-induced killing of CTCL cells is PERK dependent.** The indicated cell lines were treated with Btz (10 nM) in the presence of increasing concentrations of the PERK inhibitor GSK2960614. Cell viability data are shown.

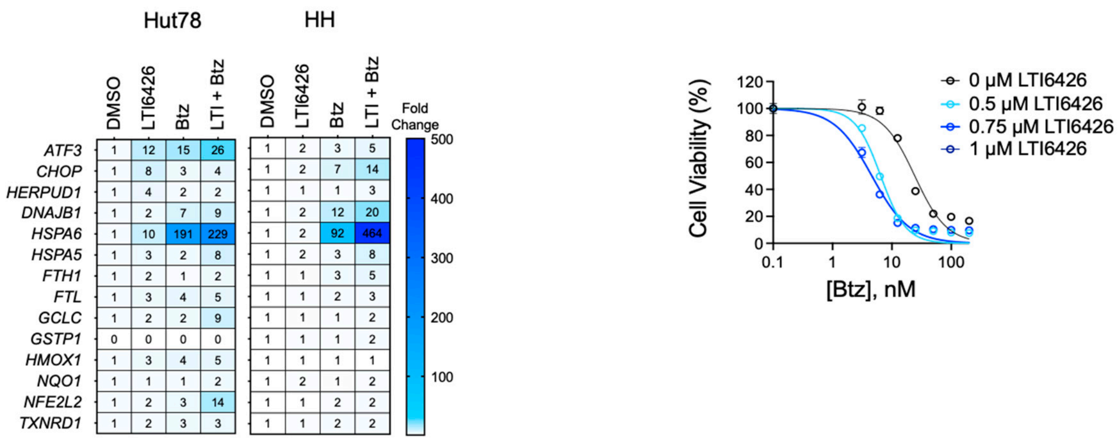


**Figure S2.** The combination of Betz and the PDI inhibitor, LTI6426, show synergistic killing of CTCL cells and induction of the *HSPA6* gene. (S2A) The indicated CTCL cells were treated with LTI6426 (500 nM), Btz (5 nM), or a combination of the two. Levels of the indicated gene transcripts were measured by RT-qPCR and shown in heat map format. Data were normalized to *GAPDH* (internal control) and DMSO (treatment control). (S2B) HH cells were treated with a dose range of Btz in the presence of the indicated concentrations of LTI6426 for 24 hours. Cell viability data are shown.

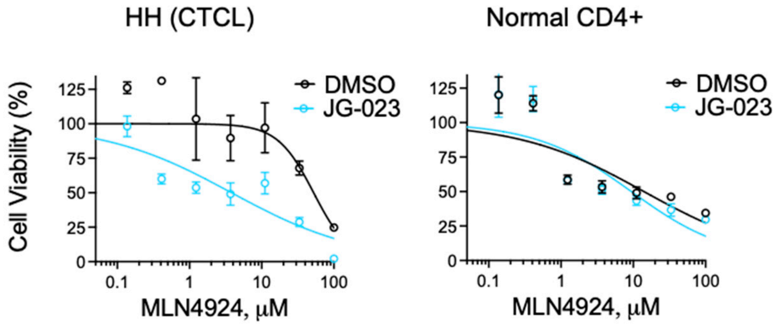


**Figure S3.** HSPA5/6 selective inhibitor, JG-023, synergistically kills CTCL cells but not normal CD4+ T cells in combination with the NAE inhibitor, MLN4924. The indicated cells were treated with a dose range of MLN4924 in the absence or presence of JG-023 (20 μM). Cell viability data are shown.

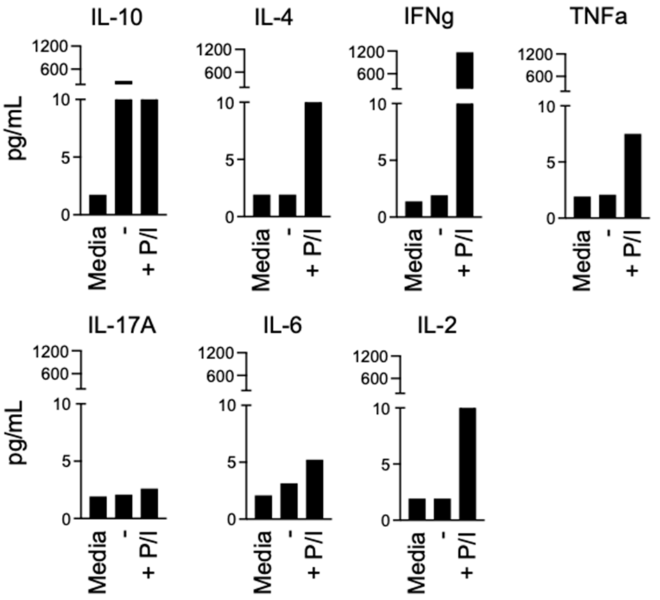


**Figure S4. CM from resting and activated CTCL show a prominent Th2 cytokine profile.** CM from resting or activated Hut78 cells was analyzed by cytokine bead array for the indicated Th1 and Th2 cytokines. Media (no cells) is shown for comparison. For activation, a combination of PMA (1 µM) and Ionomycin (200 ng/mL) was used.

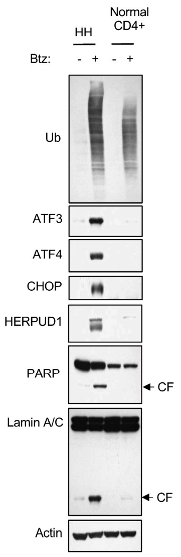


Figure 1E

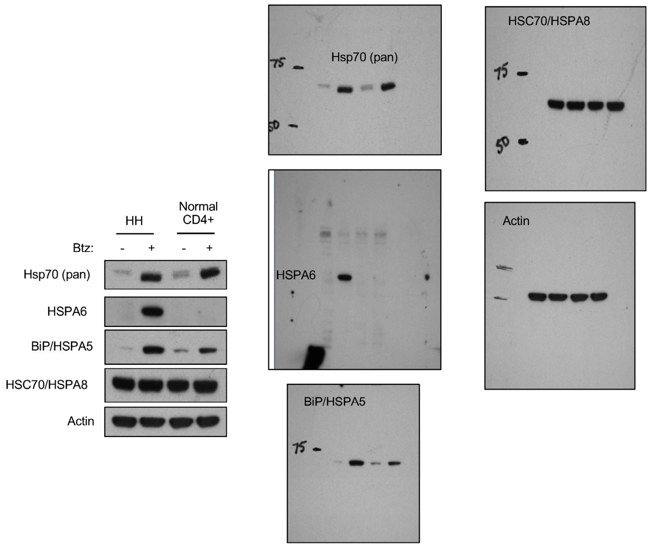


Figure 2D

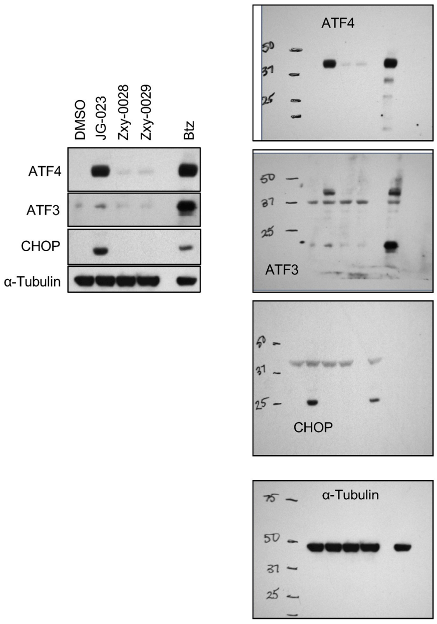


**Figure 4A**

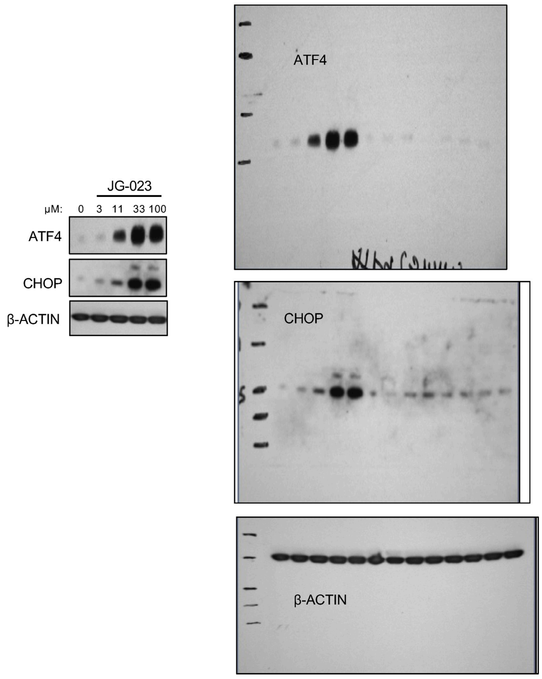


**Figure 4B**

**Figure S5.** Original images of Western Blotting figures: Figure 1E; Figure 2D; Figure 4A and 4B.
